# Response time and response time variability as indicators of response quality during static automated perimetry

**DOI:** 10.1007/s00417-021-05349-z

**Published:** 2021-09-13

**Authors:** Judith Ungewiss, Hanspeter A. Mallot, Ulrich Schiefer

**Affiliations:** 1 Competence Center Vision Research, Study Course Ophthalmic Optics, Aalen University of Applied Sciences, Anton-Huber-Str. 23, 73430 Aalen, Germany; 2 Institute of Neurobiology, Department of Biology, Tuebingen University, Tuebingen, Germany; 3 Department of Ophthalmology, Tuebingen University, Tuebingen, Germany

**Keywords:** Perimetry, Response quality, Response time, Response time variability, Catch trials

## Abstract

**Purpose:**

Perimetry is a both demanding and strenuous examination method that is often accompanied by signs of fatigue, leading to false responses and thus incorrect results. Therefore, it is essential to monitor the response quality. The purpose of this study was to evaluate the response time (RT) and its variability (RTV) as quality indicators during static automated perimetry.

**Methods:**

Size III Goldmann stimuli (25.7′) were shown with the OCTOPUS 900 perimeter in four visual field locations with 13 different stimulus luminance levels (0.04–160 cd/m^2^). An increased rate of false-positive and false-negative catch trials (25% each) served to monitor the response quality simultaneously together with response time recording. Data evaluation was divided into global and individual analysis. For global analysis, the agreement indices (AI, agreement between time periods with an increased number of false responses to catch trials *and* time periods with pathological response to time-based values set into relation to time periods in which only one of the two criteria was considered pathological) and for individual analysis, the Spearman correlation coefficients were calculated. Ophthalmologically normal subjects with a visual acuity ≥ 0.8, and a maximum spherical/cylindrical ametropia of ± 8.00/2.50 dpt were included.

**Results:**

Forty-eight subjects (18 males, 30 females, age 22–78 years) were examined. The total number of false responses to catch trials was (median/maximum): 6/82. RT and RTV were compared to the occurrence of incorrect responses to catch trials. The resulting individual Spearman correlation coefficients (median/maximum) were for RT: *ρ*_RT_ = 0.05/0.35 and for RTV: *ρ*_RTV_ = 0.27/0.61. The global analysis of the RTV showed agreement indices (median/maximum) of AI_RTV_ = 0.14/0.47.

**Conclusions:**

According to this study, an increased portion of catch trials is suitable as a verification tool for possible response quality indicators. The RTV is a promising parameter for indicating the response quality.

**Supplementary Information:**

The online version contains supplementary material available at 10.1007/s00417-021-05349-z.


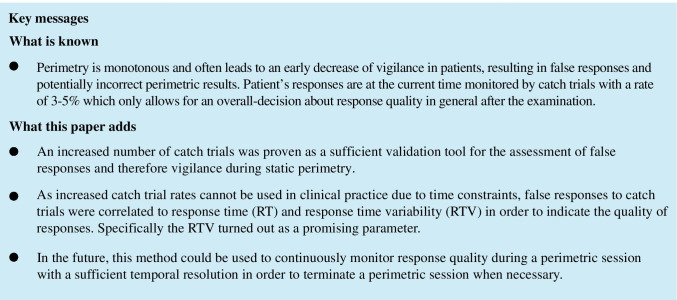


## Introduction

Perimetry is a strenuous examination method that often leads to an increasing lack of concentration and accompanying fatigue in patients, resulting in false responses and potentially incorrect perimetric results. It is therefore advisable to monitor the quality of patient’s responses continuously during perimetry.

The visual field is the entirety of visual sensory impressions that can be perceived when looking straight ahead without head and body movements [1]. The examination of the visual field within a cupola is called perimetry.

Continuous quality assessment during perimetry was first introduced by Heijl and Krakau via fixation monitoring [2, 3].

The quality of visual field examinations is nowadays usually recorded by the stability of a central fixation in combination with the number of false responses to so-called catch trials [4–6]. Due to time constraints, under clinical conditions usually a rate of only 3–5% of all stimuli is implemented as catch trials. A distinction is made between false-negative catch trials (those with highly supra-threshold, i.e. very high luminance levels which the patient would have to perceive at the corresponding normal visual field location) and false-positive catch trials (exclusive auditory stimuli without any visual stimulus presentation at that time).

The difficulty in assessing the quality of a visual field is that differential light sensitivity (DLS) fluctuates strongly: It is usually quite high at the beginning of a visual field examination but decreases during the course of a visual field examination due to decreasing vigilance [6, 7].

The term *vigilance* is frequently used synonymously with the term “alertness” and describes the degree of central nervous activation [8, 9]. This is subject to daytime variations and is usually higher in the morning and afternoon than at night [10]. Reduced vigilance is known daytime sleepiness [11]. In contrast to this, the term *fatigue* characterizes a lack of willingness to take up or sustain activities [12]. The fatigue status cannot be measured objectively, but only assessed subjectively [13]. In perimetry, fatigue is usually referred to as a decrease in visual sensitivity caused by the duration of a test procedure [14, 15].

Reaction time depends on the stimulus luminance level and is shorter for bright than for dim stimuli. This applies for normal as well as for defected areas [16, 17].

It has been reported by Surwillo and Quilter that reaction time is directly correlated to the vigilance level. In their experiment, vigilance was quantified by the percentage of pop-out stimuli being detected correctly in a visual attention-based experiment [18]. To the authors’ knowledge, there are currently no studies on the correlation between vigilance, response quality and response time and its variability during perimetry.

The aim of this study is therefore on the one hand to present a method with the help of which fatigue can be generated in a standardized, dosed form and its effect can be recorded and on the other hand to evaluate the response time (RT) and response time variability (RTV) as indicators of response quality during static automated perimetry.

## Study design and methodology

### Experimental setup

The method of constant stimuli (MoCS), where A defined number of stimuli per defined luminance level is shown at each pre-defined location in randomized order [19] was used to determine DLS with the OCTOPUS 900 perimeter (Haag-Streit AG, Koeniz, Switzerland). OPI (Open Perimetry Interface [20]) was used to implement the test algorithms: The stimulus luminance was varied in 13 equal steps between 0.04 (39 dB) and 160 cd/m^2^ (3 dB) with a background luminance of 10 cd/m^2^. Goldmann stimuli size III (25.7′) were presented 20 times each at three locations (− 6.1°, − 3.5°), (0°,7°), (6.1°, − 3.5°) and twice each at a reference location (0°, 0°) as an additional fixation stimulus for every luminance step. The examination included a total of 1612 stimuli within a time period of about 45 min. Stimuli were presented for 200 ms with an interstimulus interval of 1500 ms. The reaction time for each stimulus was monitored. Reaction time was defined as the time period between the presentation of a stimulus and response of the patient given by pressing the response button. An increased rate of false-positive and false-negative catch trials was interspersed (25% each in contrast to a total share of 3–5% under conventional clinical conditions). False-positive catch trials were defined as 0 dB stimuli (320 cd/m^2^) and false-negative catch trials were defined as 40 dB stimuli (0.032 cd/m^2^).

### Subject sample

Forty-eight subjects, equally distributed among three age groups (21–40 years, 41–60 years, 61–80 years) were included in the study. Twenty-four dominant and 24 non-dominant eyes were randomly selected. The handedness of the subjects was not evaluated. They were allowed to take the hand they preferred for operating the response button and to change hands as often as desired.

Inclusion criteria were:

- Minimum distant visual acuity (with or without correction) ≥ 0.8 (single optotypes [numbers], VISUCAT, argus individuell optic GmbH, Ottobrunn, Germany)
- Maximum spherical/cylindrical ametropia of ± 8.00/2.50 dpt
- Normal ophthalmological and general health status (determined by means of an ophthalmological examination and medical history – for more information in terms of the medical history sheet and the standard examination diagnosis sheet see Online Resource 1 and Online Resource 2)
- Signed informed consent of all tested subjects

The recruitment process was as follows:

- 54 subjects of who had completed another study earlier were addressed. 23 of them agreed to take part in the present study.
- 28 more subjects (mostly employees of the Aalen University of Applied Sciences) were recruited directly for the purpose of this study, all of whom agreed to take part.
- Three subjects had to be excluded (see "Results" chapter).

The study was approved by the Ethics Committee of the State Medical Association of Baden-Wuerttemberg and all volunteers were insured during their presence and travel to and from their home town.

### Data evaluation

Data evaluation was conducted using MatLab (Release 2018a, The MathWorks Inc., Natick, USA).

As the included parameters did not generally show a Gaussian distribution (tested with Shapiro–Wilk test, see "Results"), parameter-free statistical tests were selected for evaluation.

All results for the response time (RT), the response time variability (RTV) and the error rate for the *individual* analysis (see below) were normalized to the value range [0;1] prior to further evaluation.

False-positive and false-negative responses to catch trials were evaluated together for further evaluation. By definition, an increased number of false responses to catch trials was assumed if more than two errors per minute (corresponding to the 95th percentile of the error rate for all test persons) occurred.

**Fig. 1:**
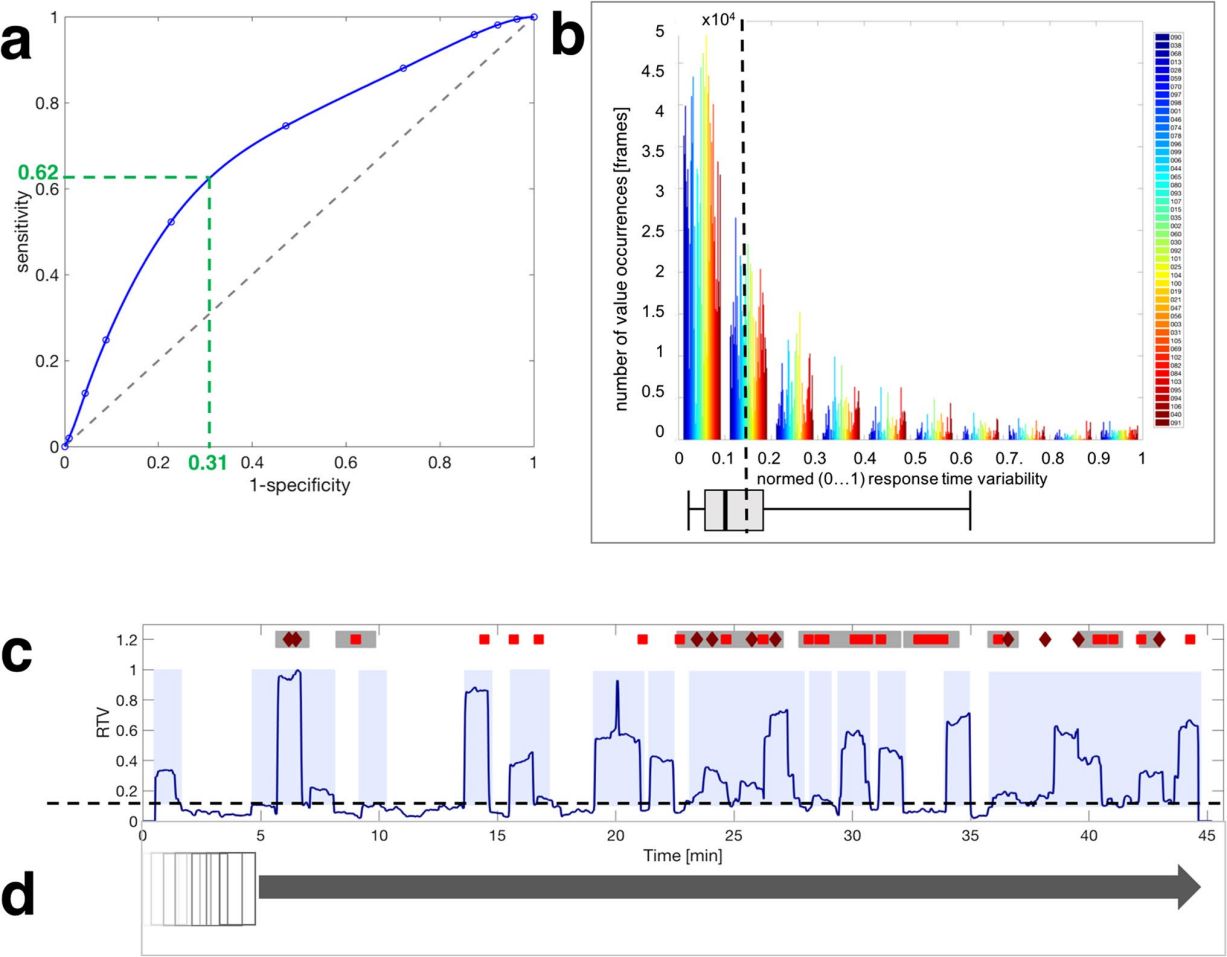
Example of the global analysis of response time variability (RTV) for subject 106. **a** The ROC curve for all subjects was calculated and “cut-off” values valid for the entire subject sample for sensitivity and specificity were determined via the Youden index (here: sensitivity = 0.62 and specificity = 0.69, see green dotted lines). **b** Using a distribution function for the RTV for the entire subject sample (the subjects were sorted in ascending order of their total number of errors, color-coded from blue: low number of errors to red: high number of errors), the specific “cut-off” value (as explained in a) corresponding to the 66th percentile was determined (here: 0.15, marked by the dashed black line). The box plot shows the distribution of all measured values for the RTV for the entire subject sample, with the thick black line indicating the median, the box marked 25th and 75th percentile and the whiskers representing 5th and 95th percentile. **c** Thus, for each subject, periods of time in which values above 0.15 (marked with the black dotted line) were present were defined as deviant values for the RTV (see light blue highlights). This figure showcases that for subject 106 as an example. Periods with an increased number of false responses to catch trials are highlighted in dark grey. The agreement index (AI) defines periods of time as congruent (and therefore as “agreement”) for time intervals with coinciding deviant values for both, the number of false responses to catch trials *as well as* for the RTV according to the global “cut-off” values evaluated as stated in **a**, **b**. In this case, the AI is 0.24. **d** Simplified graphical illustration of the method of a “sliding window”: Data within a windows of 60 s are evaluated together. The window “slides” over the complete data set frame by frame

For response time (RT) and response time variability (RTV) a linear interpolation was performed for those time phases, where no response time values were available due to the presentation of infra-threshold stimuli and correspondingly absent responses. The RTV was calculated as variance of the RT over a 60s- “sliding window” (for a simplified graphical illustration of the “sliding window” technique, see Fig. 1d).

First a *global analysis* (using the same criteria for all subjects) was performed, followed by an *individual analysis* (using individual criteria for each subject separately).

For the global analysis, sensitivity and specificity for RT and RTV were each calculated for the whole group of subjects, which means, that all RT and RTV values collected for all subjects were evaluated together for different possible cut-off values (to distinguish between measured values considered deviant or normal). The area under the ROC (receiver operating characteristics, for an overview over ROC analysis see for instance [21]) curve (AUROC) was used to determine which of the two methods was more suitable for data evaluation. Afterwards, the maximum value of the Youden index [22] was evaluated and used for the selection of the optimum cut-off values. The percentile corresponding to the optimum cut-off values was calculated.

Subsequently, a so-called agreement index (AI) was determined: The agreement between time periods with an increased number of false responses to catch trials (which was considered the gold standard) and time periods with deviant response time-based values were set into relation to time periods in which only one of the two criteria was considered pathological. The AI was calculated as follows:

$$\text{AI} = \frac{\text{increased number of false responses to catch trials AND deviant RT-based values}}{\text{increased number of false responses to catch trials OR deviant RT-based values}}$$

A representative example for this procedure is shown in Fig. 1.

For *individual analysis*, the number of false responses to catch trials was calculated “"pseudo-continuously” and thus converted into an error rate per minute, which could be correlated to RT and RTV using Spearman rank correlation analysis. Since the correlations were carried out separately for each subject, *individual* effects could be taken into account, indicating fatigue and/or sleepiness. The evaluation method with the highest median correlation coefficients was selected for the final analysis.

## Results

Each subject was given an ID between 001 and 107. For organizational reasons, not every possible number was assigned. Fifty-one numbers were assigned to potential subjects, all of whom underwent a preliminary ophthalmological-optical examination. Two of the test persons examined had to be excluded (reasons: strabismus (1), suspicion of optic nerve disease (1)). One subject ended his participation in the study after the preliminary examination.

Data sets of 48 test persons (18 male, 30 female, age 22–78 years, median age 47 years) were collected and analyzed. In one case (subject 044), the data collection stopped after some time for unknown reasons; the data available to the timepoint were nevertheless analyzed as well.

### Parameter distribution

**Fig. 2:**
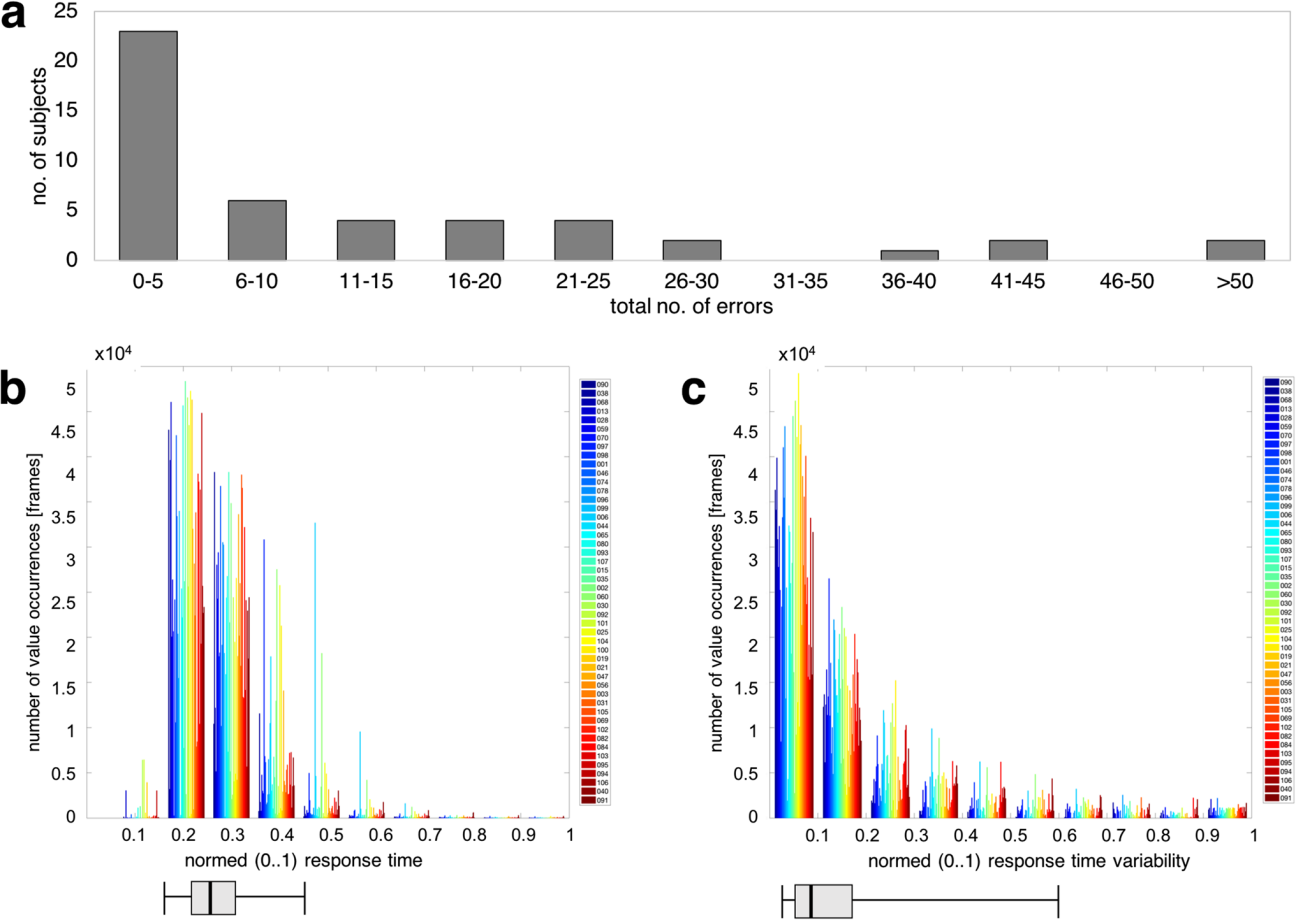
Frequency distributions of **a** false responses to catch trials (total number of errors per subject), **b** normalized (0..1) response time (RT) data (the subjects are shown in ascending order of the total number of errors. A box plot has been added, for which the following applies: box: 25th and 75th percentile, black line within the box: median, whisker: 5th and 95th percentile), **c** normalized (0..1) response time variability (RTV) data (the subjects are shown in ascending order of the total number of errors. A box plot has been added for which the following applies: box: 25th and 75th percentile, black line inside the box: median, whisker: 5th and 95th percentile)

The frequency distribution of data set was analyzed. False responses to catch trials were considered as the gold standard for the quality of responses in this study (see Fig. 2a). Data were not normally distributed (Shapiro–Wilk test, *W* = 0.69599, *p* = 1.13 × 10^−8^).

For this reason, non-parametric tests were selected for further evaluation. The median absolute RT for all subjects was 355 ms with an interquartile range (IQR) of 110 ms. For normalized RT and RTV, histograms were created to show the distribution of individual values for all subjects together (see Fig. 2b, c). As can be derived qualitatively from Fig. 2b, c, RT and RTV are also not normally distributed.

The total number of false responses to catch trials during each of the entire 45-min examinations was (median/maximum): 6/82. Here and from now on, results are displayed in this manner (median/maximum), as many patients had only a low number of false responses to catch trials. The method presented in this paper shows its strength when there are high numbers of errors—to illustrate this, in addition to the median, no measure of dispersion is given, but rather the maximum.

### Global analysis

**Fig. 3:**
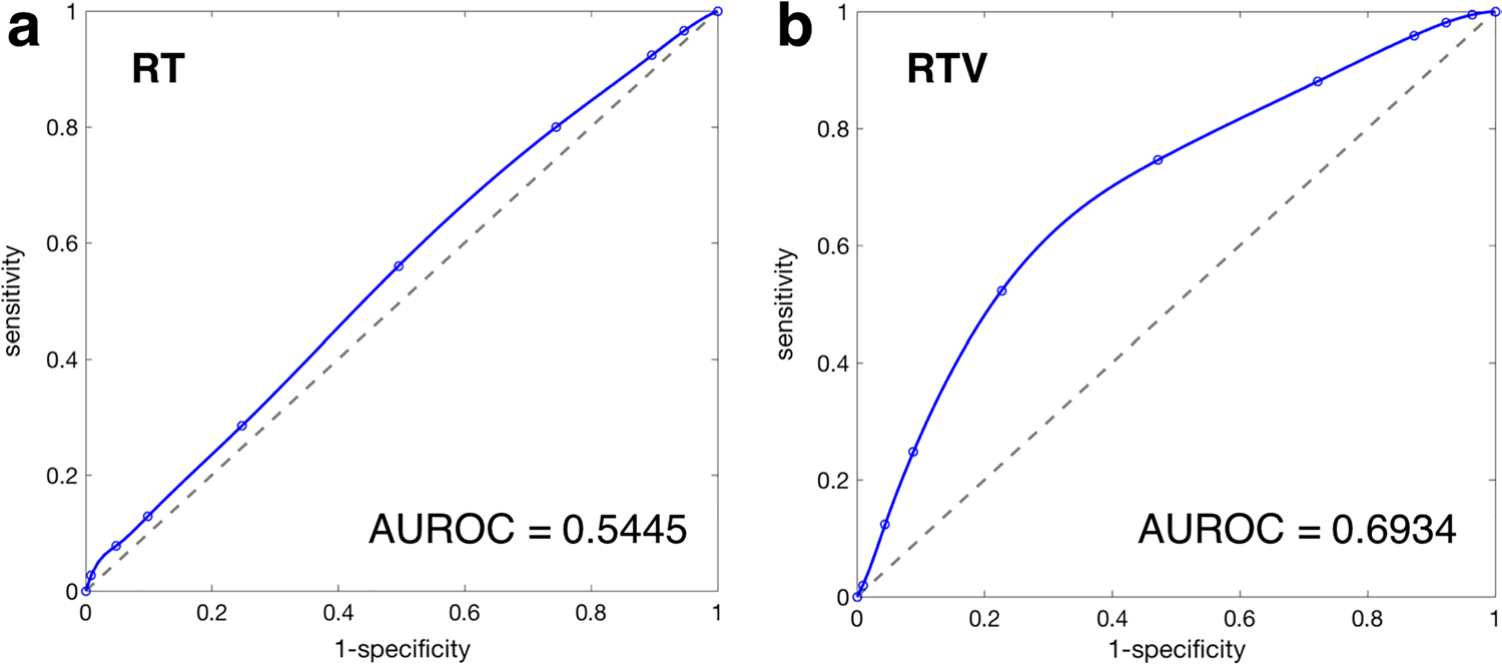
ROC (receiver operating characteristics) curves and corresponding values for the area under the ROC curve (AUROC) for **a** response time (RT) and **b** response time variability (RTV)

The ROC curves for RT and RTV are shown in Fig. 3. Since the AUROC for the RTV (AUROC = 0.6934) was larger than for the RT (AUROC = 0.5445), the RTV appeared to be a more suitable parameter for indicating the response quality during static, automated perimetry.

Cut-off values for sensitivity (0.62) and specificity (0.69) were calculated and corresponded to the 66th percentile of all measured RTV values (as can also be seen in Fig. 1).

**Fig. 4:**
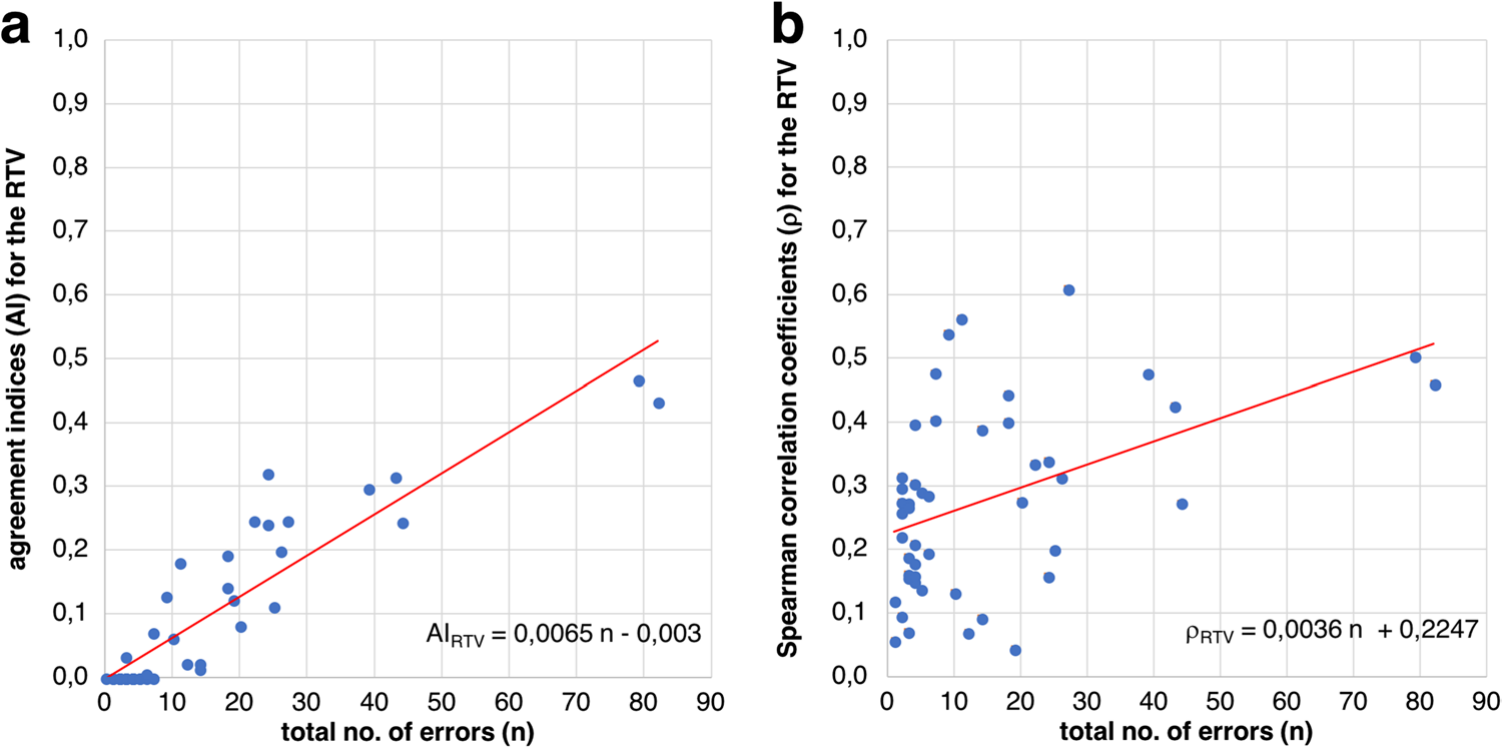
**a** Agreement indices (AI_RTV_) for the response time variability (RTV) of the global analysis as a function of the total number of errors (*n*), red line: linear regression line, **b** Individual Spearman correlation coefficients (*ρ*_RTV_) for the RTV of the individual analysis as a function of the total number of errors (*n*)

Agreement indices (AI) were calculated. Figure 4a shows the AIs of the individual subjects as a function of the total number of errors. The median for those subjects who had periods with an increased number of false responses to catch trials was AI_med_ = 0.14, the maximum value was AI_max_ = 0.47.

Figure 4a shows a “meta-correlation” between AI and the total number of errors. A correlation analysis revealed a Spearman correlation coefficient of *ρ*_global_ = 0.91 (p = 1.06 × 10^−19^).

### Individual analysis

Figure 4b shows the individual Spearman correlation coefficients as a function of the total number of errors.

Individual Spearman correlation coefficients (median/maximum) of RT: *ρ*_RT_ = 0.05/0.35 and RTV: *ρ*_RTV_ = 0.27/0.61 (*p*_max_ < 10^−4^) were obtained. The RTV thus proved to be the more suitable parameter for individual analysis.

In addition, as for the global analysis, a “meta-correlation” was performed between the individual Spearman correlation coefficients and the total number of errors per subject. This resulted in a Spearman correlation coefficient of *ρ*_individual_ = 0.45 (*p* = 1.33 × 10^−3^) and thus a medium, significant correlation.

Similar to the AIs, it was shown that correlations (albeit with lower correlation coefficients) could already be found for subjects with a low total number of errors.

## Discussion

It is known that the circadian rhythm and thus the time of day influence vigilance [10]. For that reason, indicators used to monitor vigilance, also vary during the day [23]. For this reason, the present study tried to distribute the subjects equally over different times of day. For organizational reasons (the examination dates had to be based on the availability of the subjects), however, it was not possible to achieve an equal distribution: All subjects were tested between 08:00 am and 06:00 pm. There was a tendency of testing more subjects in the morning or early afternoon than in the evening. In particular, potential influencing factors such as age or gender could not always be balanced with regard to an equal distribution throughout the day.

In perimetry, rates of 3–5% for false-positive and false-negative catch trials are common [6]. This approach thus allows for a “post-hoc” quality control, but its temporal resolution is insufficient to capture the onset of a vigilance incident. Using a rate of 4%, a stimulus duration of 200 ms and an inter-stimulus interval of 1500 ms, a catch trial is presented approximately every 21 s. Assuming that not every catch trial is necessarily answered incorrectly when fatigue sets in, a rate of 4% (or even lower) does not seem promising in terms of adequate temporal resolution of fatigue detection. Larger glaucoma or ocular hypertension studies conducted in the past have also used catch trials to test the quality of responses. Some examples are the Advanced Glaucoma Intervention Study (AGIS) [24], the Collaborative Initial Glaucoma Treatment Study (CIGTS) [25] or the Ocular Hypertension Treatment Study (OHTS) [26–28], each with a 3% catch trial rate. These catch trial rates do not appear to be sufficient for quality control with sufficient temporal resolution. The present study therefore used a rate of 25% each. The rate implemented in this study leads to a catch trial about every three seconds.

For vigilance assessment during perimetry or campimetry, efforts have been made to monitor or predict errors using pupillography. It has been shown that fatigue waves occur more frequently with increasing examination duration and that the probability of stimulus perception was higher with low amplitudes of pupillary fatigue waves [29]. However, this study was conducted using a small sample size (*n* = 13) of glaucoma patients or suspects with limited age range (51–88 years).

An attempt to predict the error rate of patients via a neural network showed a correlation of 0.72 ± 0.17 [30]. However, the correlation coefficients were unstable in comparison between patients, which was shown by the fact that some patients had positive and others negative correlation coefficients. Moreover, only nine structurally similar subjects were included in the study.

Specific questionnaires, such as the Stanford Sleepiness Scale (SSS) [31] and the Epworth Sleepiness Scale (ESS) [32]—with the latter one being the only questionnaire concerning sleepiness being validated in German language to the authors’ knowledge—are often used to assess vigilance, which is directly linked to reaction time [18]. Such questionnaires cannot be time-matched but only serve as an “overall” parameter that can be used for an estimation whether vigilance restrictions could occur during an examination in advance or retrospectively. The method presented in this paper is—in contrast—able to induce monotony in a highly standardized manner and to evaluate its effects with high time resolution.

Response times increased with decreasing vigilance. However, the RTV was even more strongly correlated to (decreasing) vigilance than the RT. According to the authors’ knowledge, the RTV has not yet been associated with vigilance. Most studies on response time examinations assume that an increased RTV is due to occasional lack of attention [33]. This could be related to a lack of concentration during exhausting tasks such as perimetry.

In contrast to the above-mentioned studies, the present study took response time and its variability into account. Both parameters can be recorded directly or indirectly during perimetry using the OCTOPUS 900 perimeter used (Haag-Streit AG, Koeniz, Switzerland). Only the present study was carried out on a representative (ophthalmologically normal) group of test persons. In direct comparison, the RTV seems to be a comparatively easy parameter to evaluate, which leads to at least comparably good results.

This study has the following weaknesses: Only ophthalmologically normal subjects were included in this study. Although each experiment lasted about 45 min, the subjects experienced considerably fewer periods of sleepiness than initially assumed. This fact had an impact on the data analysis: In all cases of missing sleepiness, no correlation with the reaction time-based parameters could be proven. With increasing total number of errors, AI and correlation coefficients between RT/RTV and error rate also increased significantly. Therefore, the method presented in this paper only shows its strength in patients with a high number of false responses to catch trials. However, as patients with an increased number of false responses to catch trials are exactly those who have to be monitored with regard to response quality, this weakness is of relatively low relevance in practice.

Regarding the global analysis of the results, it must be noted that the “cut-off” values were determined empirically using the respective distribution functions.

In summary, an increased number of catch trials was proven as a sufficient validation tool for the assessment of false responses during static perimetry. The RTV turned out as a promising parameter for indicating the quality of responses—both in global and individual terms. The results of the present study indicated that an individual consideration seemed more promising overall.

### Outlook

Patients with advanced visual field defects or neurological diseases could be included in a follow-up study. These patients would generally be expected to have a higher rate of false responses to catch trials [34, 35]. If such patients were included, the resulting “prevalence enrichment” would probably lead to a stronger correlation for the selected subgroups.

Perimeters that are currently commercially available are capable of recording response times. The RTV could be calculated internally during ongoing perimetry and thus serve as an additional quality parameter, indicating that a continuation of this investigation is not expected to produce psychophysically usable measurement results, but rather represents a vigilance test. For this purpose, an individual baseline would have to be recorded for each patient (e.g. during the first 1–2 min of an examination), to which all other values would then be assessed in relation. As soon as certain limits are exceeded, the examiner is informed to alert the patient or to terminate this perimetric session.

## Supplementary Information

Below is the link to the electronic supplementary material.

Supplementary file 1 (PDF 394 kb)

Supplementary file 2 (PDF 51 kb)

## Data Availability

The results presented in this paper are part of the dissertation “Assessment of vigilance and response quality during static automated perimetry. A study using the method of constant stimuli (MoCS) and an enhanced presentation rate of catch trials” by Judith Ungewiss. All data and material are presented and available in this dissertation.

## Declarations

### Other

The content of this paper is part of the dissertation “Assessment of vigilance and response quality during static automated perimetry. A study using the method of constant stimuli (MoCS) and an enhanced presentation rate of catch trials” by Judith Ungewiss. This topic was presented at the DOG 2020 Congress with the title “Die Reaktionszeit und deren Variabilität als Prädiktor der individuellen Antwortqualität während statischer, automatischer Perimetrie”.
