# Supplementary material for: Response time and response time variability as indicators of response quality during static automated perimetry
Source: Graefes Arch Clin Exp Ophthalmol. 2021 Sep 13;260(3):927–35. doi: 10.1007/s00417-021-05349-z (PMC8850211; doi:10.1007/s00417-021-05349-z)
Supplement: Supplementary file 1 — (PDF 394 kb) [file 417_2021_5349_MOESM1_ESM.pdf]

Hochschule Aalen

Studien-ID: ☐☐☐ Probanden-ID: ☐☐☐
Geschlecht: ☐m ☐w Alter: ________
Untersuchungsdatum: ☐☐.☐☐.☐☐☐☐
Untersucher:________________________

Anamnesebogen

Augenanamnese	Ja	Nein	Falls ja, ab welchem Lebensjahr bzw. wann wurde Erstdiagnose gestellt?	Welches Auge ist betroffen? R/L	Anmerkungen
1. Brillenträger?	☐	☐	☐☐. Lj.		____________ ____________
2. Kontaktlinsenträger?	☐	☐	☐☐. Lj		____________ ____________
3. Sehen Sie auf beiden Augen mit Korrektion gleich gut?	☐	☐			____________ ____________
4. Schielen bekannt?	☐	☐	☐☐. Lj	R ☐ L ☐	____________ ____________
5. Sind Sie schwachsichtig? (Amblyopie)?	☐	☐	☐☐. Lj	R ☐ L ☐	____________ ____________
6. Augenbewegungsstörungen bzw. Doppelbilder?	☐	☐			____________ ____________
7. Grauer Star (Katarakt)?	☐	☐	☐☐. Lj	R ☐ L ☐	____________ ____________
8. Grüner Star (Glaukom)?	☐	☐	☐☐. Lj	R ☐ L ☐	____________ ____________
9. Netzhauterkrankungen? (z.B. Netzhautablösung,..)	☐	☐	☐☐. Lj	R ☐ L ☐	____________ ____________
10. Makulaerkrankungen?	☐	☐	☐☐. Lj	R ☐ L ☐	____________ ____________

Hochschule Aalen

Studien-ID: ☐☐☐ Probanden-ID: ☐☐☐
Geschlecht: ☐m ☐w Alter: ________
Untersuchungsdatum: ☐☐.☐☐.☐☐☐☐
Untersucher:________________________

**Augenanamnese**	**Ja**	**Nein**	**Falls ja, ab welchem Lebensjahr bzw. wann wurde Erstdiagnose gestellt?**	**Welches Auge ist betroffen? R/L**	**Anmerkungen**
11. Schwere chronische Augenentzündungen?	☐	☐	☐☐. Lj	R ☐ L ☐	____________________<br>____________________
12. Augenverletzungen?	☐	☐	☐☐. Lj	R ☐ L ☐	____________________<br>____________________
13. Augenoperationen?	☐	☐	☐☐. Lj	R ☐ L ☐	____________________<br>____________________
14. Augenmedikamente? (Tropfen/Salben)	☐	☐	☐☐. Lj	R ☐ L ☐	____________________<br>____________________
15. Sind in Ihrer Familie Augenerkrankungen bekannt? (z.B. hohe Fehlsichtigkeiten, Glaukom [Grüner Star], Katarakt [Grauer Star], Netzhautablösung, Farbsehstörungen,….)	☐	☐			____________________<br>____________________<br>____________________<br>____________________<br>____________________<br>____________________

**Allgemeinanamnese**	**Ja**	**Nein**	**Falls ja, ab welchem Lebensjahr bzw. wann wurde Erstdiagnose gestellt?**	**Anmerkungen**
1. Herz-Kreislauferkrankung?	☐	☐	☐☐. Lj	____________________<br>____________________
2. Andere Organerkrankung? (z.B. Leber, Niere, Magen,..)	☐	☐		____________________<br>____________________
3. Neurologische Erkrankungen? (z.B. Schlaganfall, Epilepsie)	☐	☐	☐☐. Lj	____________________<br>____________________

Studien-ID: ☐☐☐ Probanden-ID: ☐☐☐
Geschlecht: ☐m ☐w Alter: _________
Untersuchungsdatum: ☐☐.☐☐.☐☐☐☐
Untersucher:_________________________

Allgemeinanamnese	Ja	Nein	Falls ja, ab welchem Lebensjahr bzw. wann wurde Erstdiagnose gestellt?	Anmerkungen
4. Stoffwechselerkrankung? (z.B. Blutzucker, Schilddrüse, Fettstoffwechsel,..)	☐	☐	☐☐. Lj	____________ ____________ ____________
5. Seelische Erkrankungen?	☐	☐	☐☐. Lj	____________ ____________
6. Schwangerschaft?	☐	☐		____________ ____________
7. Medikamenteneinnahme?	☐	☐	☐☐. Lj	____________ ____________
8. Medikamente, die die Reaktionszeit beeinflussen?	☐	☐	☐☐. Lj	____________ ____________
9. Andere Erkankungen?	☐	☐	☐☐. Lj	____________ ____________
10. Haben Sie Allergien?	☐	☐		____________ ____________

Sonstige Anmerkungen:

____

____
