# Supplementary material for: Response time and response time variability as indicators of response quality during static automated perimetry
Source: Graefes Arch Clin Exp Ophthalmol. 2021 Sep 13;260(3):927–35. doi: 10.1007/s00417-021-05349-z (PMC8850211; doi:10.1007/s00417-021-05349-z)
Supplement: Supplementary file 2 — (PDF 51 kb) [file 417_2021_5349_MOESM2_ESM.pdf]

Hochschule Aalen

Studien-ID: ☐☐☐ Probanden-ID: ☐☐☐

Geschlecht: ☐m ☐w Alter: ________

Untersuchungsdatum: ☐☐.☐☐.☐☐☐☐

Untersucher:______________________

Untersuchungsprotokoll

Augenstellung und Augenbeweglichkeit

	RA Intakt/Unauffällig	RA Gestört/Auffällig	LA Intakt/Unauffällig	LA Gestört/Auffällig	Bemerkungen
Augenstellung	☐	☐	☐	☐	
Augenbeweglichkeit	☐	☐	☐	☐	

Pupillen (Efferenz und Afferenz)

Pupillen	Ja	Nein	RA auffällig	LA auffällig	
Pupillen rund	☐	☐	☐	☐	
Pupillen zentriert	☐	☐	☐	☐	
In Helligkeit und Dunkelheit isokor?	☐	☐			
- Anisokorie?					☐ R > L ☐ R < L
- Bei Dunkelheit zunehmend?	☐	☐			Dilatationsdefizit
- Bei Helligkeit zunehmend?	☐	☐			Kontraktionsdefizit

RAPD?	☐	☐	☐ __ , __ logE	☐ __ , __ logE	
Sonstige Anmerkungen					

Vorderer Augenabschnitt

Spaltlampe (WQ900, Haag Streit, Köniz)

	RA Intakt/Unauffällig	RA Gestört/Auffällig	LA Intakt/Unauffällig	LA Gestört/Auffällig	Bemerkungen
Bindehaut					
Hornhaut	☐	☐	☐	☐	
Vorderkammer	☐	☐	☐	☐	
Vorderkammertiefe temporale Peripherie (Grad 1-4, nach W. van Herick)	☐ 0 ☐ 1 ☐ 2	☐ 3 ☐ 4	☐ 0 ☐ 1 ☐ 2	☐ 3 ☐ 4	
Irisfarbe					
Iris/Pupille	☐	☐	☐	☐	
Linse	☐	☐	☐	☐	
Glaskörper	☐	☐	☐	☐	
Intraokular reizfrei?	☐	☐	☐	☐	

Augenhintergund

BETA 200 S LED Ophthalmoskop, Heine, Herrsching

	RA		LA	
	Intakt/Unauffällig	Gestört / Auffällig	Intakt/Unauffällig	Gestört / Auffällig
Papille randscharf?	☐	☐	☐	☐
Papille vital gefärbt?	☐	☐	☐	☐
Zentrale Exkavation?	☐	☐	☐	☐
CDR:				
ISNT Regel erfüllt?	☐	☐	☐	☐
Papillenrand / Nervenfaserschicht	☐	☐	☐	☐
Makula?	☐	☐	☐	☐
	Ausprägungsgrad		Ausprägungsgrad	
Wallreflex	☐ + ☐ (+)	☐ ø	☐ + ☐ (+)	☐ ø
Foveolarreflex	☐ + ☐ (+)	☐ ø	☐ + ☐ (+)	☐ ø
	Intakt/Unauffällig	Gestört / Auffällig	Intakt/Unauffällig	Gestört / Auffällig
Zentrale Fixation?	☐	☐	☐	☐
Exzentrischer Fixationsort:				
Gefäße?	☐	☐	☐	☐
a : v =				
Peripherie?	☐	☐	☐	☐
**Bemerkungen**				
